# Protective effect of resveratrol against light-induced retinal degeneration in aged SAMP8 mice

**DOI:** 10.18632/oncotarget.19473

**Published:** 2017-07-22

**Authors:** Zhirong Liu, Zhengzheng Wu, Jie Li, Anna Marmalidou, Ruifan Zhang, Man Yu

**Affiliations:** ^1^ Department of Ophthalmology, Hospital of University of Electronic Science and Technology of China and Sichuan Provincial People’s Hospital, University of Electronic Science and Technology of China, Chengdu, Sichuan 610072, China; ^2^ Schepens Eye Research Institute, Massachusetts Eye and Ear Infirmary, Harvard Medical School, Boston, MA 02114, USA

**Keywords:** resveratrol, aging, SAMP8 mice, light damage, retinal degeneration

## Abstract

**Purpose:**

The purpose of this study was to determine the protective effects of Resveratrol (RESV) on acute bright light-induced retinal degeneration in aged senescence accelerated mouse strain.

**Methods:**

Ten three-month-old male SAMP8 mice (prone to aging) were randomly assigned to two experimental dietary groups: one untreated group and one RESV treatment group (n=20 eyes for each group). After 30 days of treatment, mice were exposed to intense bright light. Ten male SAMR1 mice (resistant to aging) served as control (n=20 eyes). The protective effects of RESV administration on light-induced retinal degeneration in SAMP8 strain as well as the effect of bright light damage in the retinas of SAMP8 mice were analyzed by electroretinography (ERG), retinal histology, mRNA, protein and lipid profile.

**Results:**

68%-85% of a-wave amplitude and 72%-92% of b-wave amplitude were persevered by RESV in SAMP8 mice that were exposed to light damage. Also, RESV preserved their photoreceptor nuclei. mRNA expression of neuroprotective factors leukemia inhibitory factor (LIF), brain derived neurotrophic factor (BDNF), oncostatin M (OSM), cardiotrophin 1(CT-1) and cardiotrophin-like cytokine (CLC) were up-regulated 28, 8, 7, 5 and 9-fold in SAMP8 mice after RESV treatment. In addition, RESV could suppress the NF-κB pathway by down-regulating the expression of pIκB. Light damage led to increase of saturated FA, monoenoic FA, n6 PUFA and n6/n3 ratio and decrease of Docosahexaenoic acid (DHA). There was no significant difference on DHA and the ratio of n6/n3-FA between the untreated and RESV treated SAMP8 mice.

**Conclusions:**

Collectively, our study provides evidence that RESV prevents light-induced retinal damage associated with aging.

## INTRODUCTION

Aging is associated with an increasing prevalence of neurodegenerative disorders and leads to irreversible alterations in humans and experimental animals [1]. The retina, a layer of nervous tissue that covers the inside of the back of the eyeball, is exposed to a variety of environmental insults and stressors, including age-associated alterations and lighting that impair its function. Light damage involves an oxidative stress that may contribute to the pathogenesis of several aging-related retinal degenerative diseases such as age-related macular degeneration (AMD) and diabetic retinopathy (DRP) [2, 3].

Resveratrol (3, 5, 4’-trihydroxy-trans-stilbene; RESV) [4], a phytoalexin naturally synthesized or induced in plants, is a widely known antioxidant and anti-inflammatory agent and has been shown to reverse age-associated pathologies in small mammals [5]. A recent study showed that administration of RESV decreases nuclear-factor kappa B (NF-κB) and reduces the proinflammatory and prooxidant status associated with aging, which restores the function of the affected pancreas of aged SAMP8 mice [6]. The senescence-accelerated prone mouse (SAM) was established from the AKR/J strain. The SAM mice include 9 strains of accelerated-senescence prone, short-lived mice (SAMP) and 3 strains of accelerated senescence-resistant, long-lived mice (SAMR), which has been extensively studied to understand age-related human diseases [7, 8]. Among SAMP mice, SAMP8 mice have been extensively used in previous studies to investigate the effect of aging in different organs as mentioned above in pancreas. Also, these aging mice are considered as a good model of Alzheimer’s disease to study the fundamental mechanisms of age-associated deficiencies in learning and memory [9]. Interestingly, a number of striking similarities between Alzheimer’s disease and AMD have been described. Both represent a complex and multi-factorial degeneration of central nervous system in which aging is a primary risk factor [10]. In addition to age-associated changes in the brain, the SAMP mice also demonstrate neuronal cell degeneration in the retina [11]. Aged SAMP8 mice have been recently shown to develop retinopathy similar to some features of human AMD including Aβ deposition and increased inflammatory response in the outer retina [12].

Age-related susceptibility of retinal photoreceptors to light-induced degeneration was strongly influenced by exposure to different intensities of environment light. Daniel and coworkers have reported significant differences in the extent of light damage in two- to twelve-month-old albino rats [13]. The aim of our study was to determine the protective effects of RESV on acute bright light-induced retinal degeneration in an aging animal model using SAMP8 mice for the first time in the study of light-induced retinal degeneration.

## RESULTS

### Functional evaluation with electroretinography (ERG)

**Figure 1:**
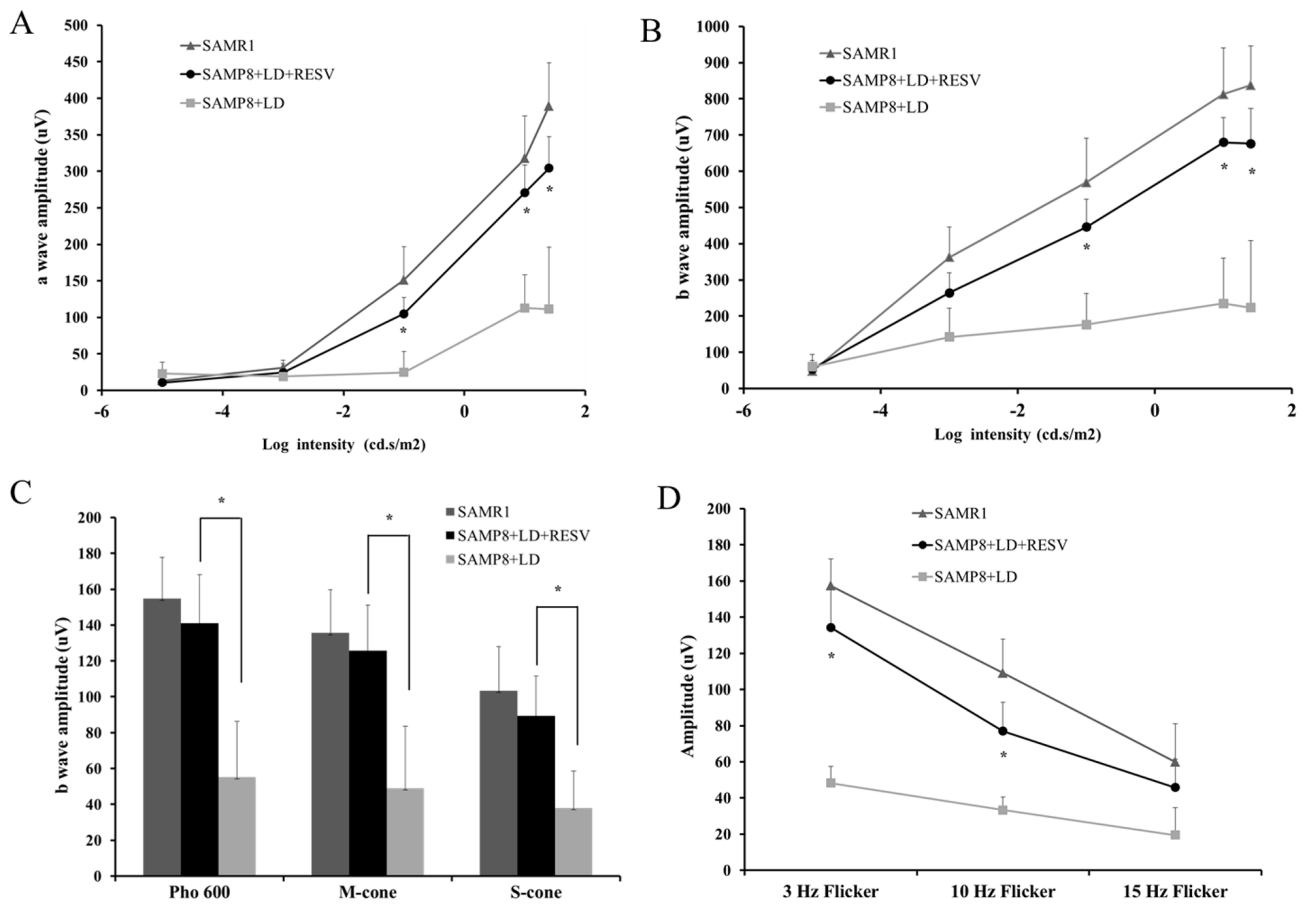
RESV prevented the rod and cone degeneration of aged SAMP8 mice from light damage **(A)** and **(B)**: scotopic d a-wave and b-wave amplitudes of SAMR1 and SAMP8 mice that have been light damaged (2,700 lux for 4h) with or without RESV supplementation; The stimulus intensities for scotopic ERG were −5 followed by −3, -1, 1and 1.2 log cd.s/m^2^. **(C)** photopic b-wave amplitude: photopic 600 lux, short-wave-sensitive cone (S-cone)-mediated and middle-wave-sensitive cone (M-cone)-mediated ERG response were recorded. **(D)** The flicker response was taken with 3Hz, 10Hz and 15Hz light flicks. n=20; value=Mean±SE (*p<0.05, vs. SAMP8+LD, paired t-test).

To understand the role of RESV in the aging retina, RESV was administered to SAMP8 mice, which were then subjected to severe light stress at 2,700 lux for 4h and returned to standard light conditions. After 7 days of recovery, ERG was performed on all mice to evaluate their retinal function. The ERG a-wave and b-wave are the major components of murine ERG recording and are comprehensively studied. It is generally accepted that the a-wave of scotopic ERG is mainly derived from rod photoreceptors in response to a flashed stimulus and cone-driven a-wave is relatively negligible under light-adapted (photopic) conditions [14]. The ERG b-wave represents the activity of cone photoreceptors and originates from the inner retinal neurons [15]. Therefore, in this study, we analyzed the changes on amplitudes of scotopic a-wave and scotopic and photopic b-wave that reflect rod and cone function respectively. As expected, the untreated SAMP8 mice that were exposed to light damage showed significantly reduced amplitude of scotopic a-wave (Figure 1A), scotopic b-wave (Figure 1B), and photopic b-wave (Figure 1C). Furthermore, the exacerbation of cone function in SAMP8 without RESV treatment was reflected by the significant reduction in the flicker ERG that originates primarily in the inner retina (Figure 1D). However, SAMP8 mice that were administered with RESV were capable to maintain their retinal function. Consequently, approximately 68%-85% of rod-driven a-wave amplitude and 72%-92% of cone-driven b-wave amplitude were persevered by RESV.

### Morphologic evaluation with quantitative histology

**Figure 2:**
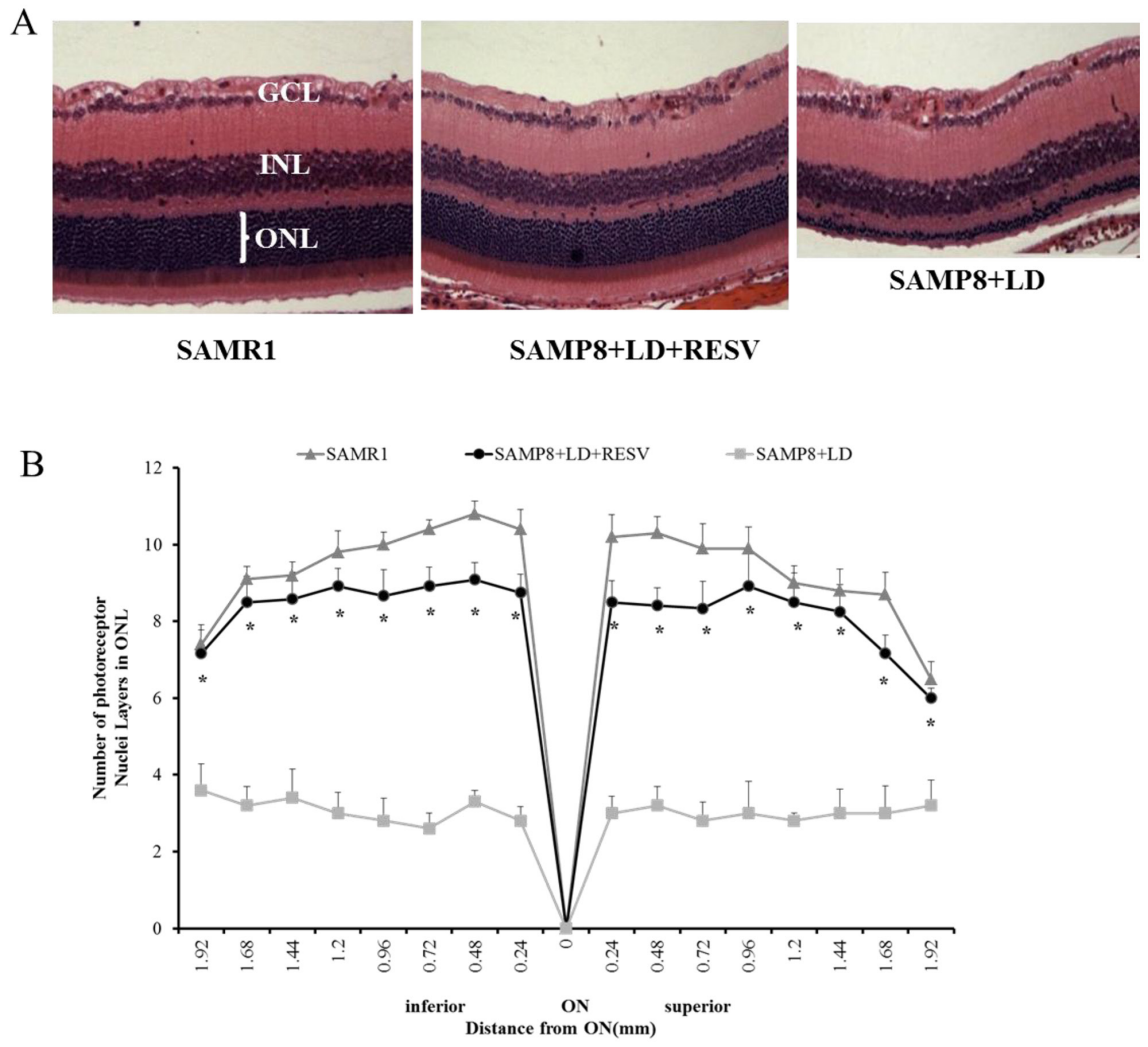
RESV preserved the structure of photoreceptor layer of aged SAPM8 mice that were exposed to light damage **(A)** Representative sections and **(B)** quantification of number of layers of photoreceptor nuclei in the outer nuclear layer (ONL) along the vertical meridian of the retina. n=10; value=Mean±SE (*p<0.01, vs. SAMP8+LD, paired t-test). ONL-Outer nuclear layer; INL-Inner nuclear layer; GCL-Ganglion cell layer; ON-Optic nerve.

After ERG analysis, we evaluated the extent of photoreceptor cell loss by quantitative histology. The number of layers of photoreceptor nuclei in the outer nuclear layer (ONL) was analyzed as described by Ueki et al [16]. Figure 2A and 2B showed representative sections of the inferior retina and quantitative alterations in photoreceptor nuclei in ONL, respectively. Compared with SAMR1 undamaged retinas that contain 10-11 layers of photoreceptor nuclei in the ONL, SAMP8 mice following light exposure without treatment retained only 2-4 layers of photoreceptor nuclei. However, while SAMP8 mice with RESV supplementation suffered from severe light stress, their retinal degeneration was mild as they retained 8–9 layers of photoreceptor nuclei. These data demonstrate that exposure to 2700lux light for 4h resulted in significant loss of photoreceptor nuclei in the ONL of the retina in the untreated group. A great preservation of photoreceptor nuclei in the RESV-treated group was observed (P<0.01, vs. SAMP8+LD).

### Expression of neuroprotective factors after RESV treatment

**Figure 3:**
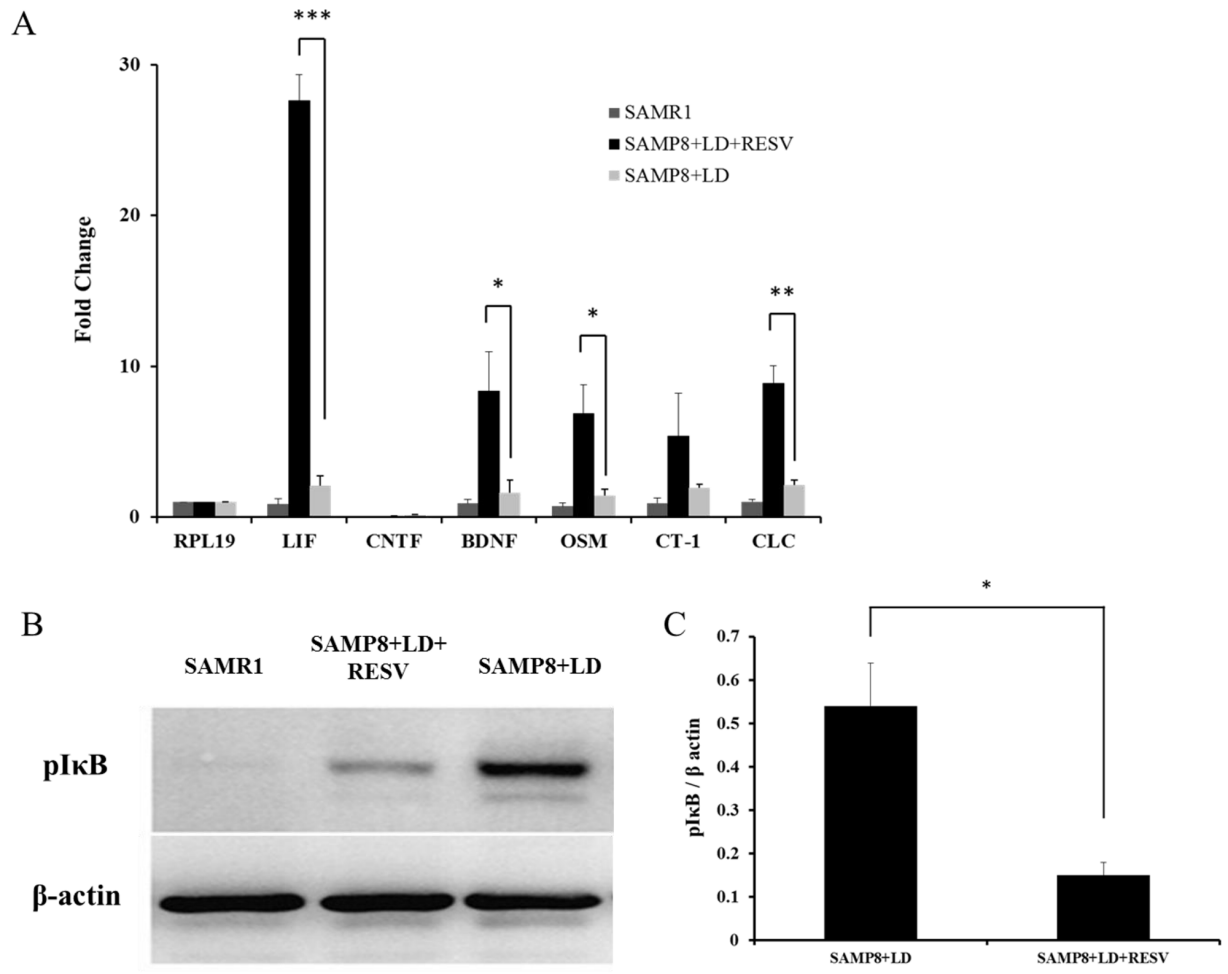
RESV supplementation leads to up-regulation of neuroprotective factors **(A)** Quantitative evaluation of cytokine mRNA expression levels using real-time PCR. Comparison of quantitative expression of different genes presented relative to the expression of the housekeeping gene RPL19. The values represent the Mean±SD (n=4) after normalizing with RPL19 calculated by the comparative threshold cycle method. LIF, BDNF, OSM, CT-1 and CLC exhibit a significant up-regulation of 28, 8, 7, 5 and 9-fold respectively in SAMP8 mice with RESV treatment (*p<0.05, **P<0.01, ***P<0.001, vs. SAMP8+LD, unpaired t-test). **(B)** Representative western blots and **(C)** Quantification of protein expression levels of pIκBα, n=3, value=Mean±SD. An increase in pIκB protein was observed in SAMP8 mice with RESV treatment. (*p<0.05, vs. SAMP8+LD, unpaired t-test).

One of the protective mechanisms of the retina to resist light damage is to regulate the levels of neuroprotective cytokines [17]. IL-6 family cytokines including leukemia inhibitory factor (LIF) [16], basic ciliary neurotrophic factor (CNTF) [18], oncostatin M (OSM) [19], cardiotrophin-1(CT-1) [20], and cardiotrophin-like cytokine (CLC) [21] have been reported to protect photoreceptors in different animal models. In addition, an increase in gene expression of brain derived neurotrophic factor (BDNF) was observed under light-induced damage in a previous study [22]. To determine which factors could contribute to the protective effect of RESV in SAMP8 mice that were exposed to light stress, levels of gene expression of those neuroprotective factors were quantified using real-time PCR. Ribosomal protein RPL19 was used as housekeeping gene. Comparison of expression of different genes relative to the expression of RPL19 (Figure 3A) showed that LIF, OSM, CT-1 and CLC were up-regulated 28, 7, 5 and 9-fold in SAMP8 mice after RESV treatment. BDNF expression exhibited an 8-fold increase. However, we did not observe any up-regulation of CNTF in SAMP8 mice.

Furthermore, it has been postulated that RESV not only curbs expression of NF-κB, but also impedes the phosphorylation of IκB (pIκB) thereby keeping the constitutive NF-κB subunit in an inactive state, resulting in suppression of the inflammatory alteration [23]. Protein levels of pIκB were measured to evaluate the inactivation of NF-κB. Figure 3B and 3C demonstrate that RESV supplementation could suppress the NF-κB pathway by down-regulating the expression of pIκB.

### Fatty acid (FA) analysis

A recent study showed that RESV exhibits the effects on the metabolic kinetics of sphingosine 1-phosphate, a multi-functional phospholipid, and that high concentration of RESV could increase LDL receptor expression [24]. We speculated that RESV might affect the metabolism of saturated and/or unsaturated FAs in the retina. Fatty acid composition in the retinas were analyzed by GC-MS. Compared to the untreated SAMP8 mice, the retinas of RESV treated mice had lower relative mole% of the 16:0 (Saturated FA), 18:1 (Monoenoic FA), 18:2n6 and 20:4n6 (n6 Polyunsaturated FA, PUFA). There was no significant difference in 20:5n3 (Eicosapentaenoic acid, EPA), 22:6n3 (Docosahexaenoic acid, DHA) and the ratio of n6/n3-FA between the untreated and RESV treated SAMP8 mice. Compared to the undamaged retinas of SAMR1 control mice, the retinas of RESV treated SAMP8 mice had higher 16:0 and 18:1, and lower DHA. Light damage to the retinas of SAPM8 mice not only led to an increase of 16:0, 18:1 and 18:2n6, but also decrease of DHA. Although the average of n6/n3-FA ratio in SAMP8+LD group was almost twice more than that of SAMP8+LD+RESV group, there was no significant difference in the n6/n3-FA ratio between those two groups. However, a significant increase of n6/n3-FA ratio was observed in the retinas of SAMP8+LD group without treatment when compared to that of SAMR1 control mice (Table 1).

**Table 1:** Relative mole percentage (±SD) of fatty acid composition from mice retina

FA	SAMP1 (a)	SAMP8+LD+RESV (b)	SAMP8+LD (c)	P value (a) vs.(b)	P value (b) vs.(c)	P value (a) vs.(c)
14:0	0.44±0.08	0.53±0.16	0.3±0.03	> 0.05	> 0.05	> 0.05
16:0	15.1±0.48	18.33±1.44	19.67±0.28	< 0.001	< 0.01	< 0.001
16:1	1.93±0.15	2.45±0.4	1.95±0.64	> 0.05	> 0.05	> 0.05
18:0	16.22±0.29	16.03±0.27	16.68±0.37	> 0.05	> 0.05	> 0.05
18:1	15.41±0.64	18.71±0.17	27.27±0.8	< 0.001	< 0.001	< 0.001
18:2n6	7.49±0.16	7.72±0.07	9.51±0.43	> 0.05	< 0.001	< 0.001
18:3n6	0.04±0.00	0.06±0.01	0.08±0.01	> 0.05	> 0.05	> 0.05
18:3n3	0.55±0.17	0.65±0.09	0.26±0.02	> 0.05	> 0.05	> 0.05
20:0	0.83±0.02	0.81±0.05	1.09±0.05	> 0.05	> 0.05	> 0.05
20:1	0.9±0.03	0.87±0.05	1.56±0.12	> 0.05	> 0.05	> 0.05
20:5n3	0.26±0.02	0.36±0.09	0.15±0.09	> 0.05	> 0.05	> 0.05
20:2n6	0.41±0.02	0.32±0.02	0.55±0.02	> 0.05	> 0.05	> 0.05
20:3n6	0.39±0.03	0.22±0.03	0.27±0.08	> 0.05	> 0.05	> 0.05
20:4n6	3.46±0.06	3.44±0.05	4.93±0.07	> 0.05	< 0.001	< 0.001
22:0	0.65±0.01	0.60±0.03	0.81±0.03	> 0.05	> 0.05	> 0.05
22:1	0.27±0.01	0.26±0.02	0.36±0.02	> 0.05	> 0.05	> 0.05
22:4n6	0.29±0.01	0.33±0.01	0.32±0.02	> 0.05	> 0.05	> 0.05
22:5n6	0.49±0.01	0.13±0.01	0.23±0.01	> 0.05	> 0.05	> 0.05
22:5n3	1.33±0.09	1.28±0.58	1.09±0.13	> 0.05	> 0.05	> 0.05
22:6n3	15.05±2.09	13.35±3.87	12.5±1.16	< 0.001	> 0.05	< 0.001
24:0	0.86±0.04	0.88±0.03	0.84±0.06	> 0.05	> 0.05	> 0.05
24:1	0.66±0.00	0.57±0.02	0.68±0.04	> 0.05	> 0.05	> 0.05
24:6n3	0.07±0.01	0.02±0.00	0.02±0.01	> 0.05	> 0.05	> 0.05
32:6n3	0.16±0.03	0.10±0.05	0.04±0.01	> 0.05	> 0.05	> 0.05
34:5n3	0.11±0.02	0.09±0.05	0.04±0.01	> 0.05	> 0.05	> 0.05
34:6n3	0.20±0.03	0.15±0.08	0.05±0.01	> 0.05	> 0.05	> 0.05
n6/ n3 ratio	0.40±0.02	0.68±0.03	1.12±0.53	> 0.05	> 0.05	< 0.05

**FA: fatty acid**

Relative mol%s (±SD, n=3) of FA from total lipids extracted from sample homogenate equivalent to 2.0 mg of protein were converted to FAME and analyzed by GC-FID. Multivariate ANOVA followed by posthoc Scheffe test was used to compare the data from the different groups.

## DISSCUSION

RESV has garnered much attention as a phytochemical, which exerts antioxidant and anti-inflammatory effects in various mammalian aging models [5, 25]. Those studies provided increasing evidence that RESV has beneficial effects in neurodegenerative diseases such as AD [26], Parkinson’s Disease (PD) [27], and Amyotropic Lateral Sclerosis (ALS) [28, 29]. Recently, protective effects of RESV have been investigated *in vitro* on retinal pigment epithelium cell death resulting from blue-light-induced damage [30], and *in vivo* on the rat model of diabetic retinopathy [31]. In this study, the effect of RESV in light-induced retinal damage of SAMP8 mice was analyzed to understand the mechanism of restoration of function of the affected retina following RESV administration in an aging animal model.

Initially, we used the intensity of 4000 lux for 6 hours that caused severe irreversible damage in the retina of SAMP8 mice and RESV couldn’t prevent this damage (data not shown). Then, we reduced the light intensity to 2,700 lux for 4 hours, which induced the morphological changes in the SMAP8 mice as we showed in Figure 2A. As described above, aging is a well-known risk factor for retinal degenerative diseases and light damage involves an oxidative process that may trigger apoptosis in the light-sensitive retina. A previous study showed that the retina in older rats sustained more damage from exposure to intense light compared to retina derived from younger ones [13]. Actually, in our study, this moderate intensity of 2,700 lux for 4 hours didn’t cause the morphological damage in the SAMR1 control mice, but the slight alterations of ERG function (data not shown), suggesting that SAMP8 mice are more susceptible to light exposure than SAMR1 mice.

Out data showed that RESV could preserve retinal structure and function of SAMP8 mice that were exposed to severe light stress. However, the underlying protective mechanisms remain unclear. It has been reported that RESV could induce neuronal differentiation of medulloblastoma cells via increased production and secretion of LIF [32]. Previous studies have demonstrated that intraocular injection of LIF and CNTF can protect photoreceptor from light damage [16, 21, 33]. In our experiments with RESV supplementation, we observed a significant up-regulation of neuroprotective factors LIF, OSM and CLC (Figure 3A). However, CNTF was not up-regulated in the retinas of SAMP8 mice in response to RESV treatment. The increase in CNTF in response to preconditioning was observed in rats, but not in mice [34, 35], which implied that the differences in neuroprotective actions of endogenous factors might be associated with species differences. Actually, we also observed that cytokine CT-1 was up-regulated 5-fold in SAMP8 mice after RESV treatment, but there was no significant difference between the untreated and treated group. In addition, up-regulation of the neuroprotective factor BDNF was observed in the retinas of the treatment group. BDNF plays an essential role in the maintenance and survival of neurons and oligodendrocytes. It was postulated that RESV can promote the release of neurotrophic factors including BDNF in the central nervous system [36]. Our findings also support that RESV could mediate the up-regulation of BDNF in the light-damaged retina.

The causes of aging are due to multi-factorial processes. It is well known that aging is associated with inflammation, NF-κB activation, increased apoptosis, impaired mitochondrial activity, and a decline in immune function, as well as increased free-radical production rate [6]. Our results showed a decrease in pIκB expression levels in SAMP8 mice with RESV treatment (Figure 3B and 3C), which is consistent with observations from previous studies [1, 6, 37]. Therefore, it is plausible that RESV can mediate anti-inflammatory and/or anti-oxidation effects via inhibiting NF-κB pathway.

In animal models, n3 PUFA supplementation has been shown to enhance [38] or prevent [39, 40] the reduction in ERG a- and b-wave amplitudes after transient ischemia. In the 1970s, Anderson and colleagues demonstrated that FA composition in rod outer segment membranes of rodent retina is an essential determinant for optimal retinal function. In addition, they showed that reduction of PUFA in mouse retinas leads to changes in retinal function measured by reduced amplitudes of their ERG [41, 42]. Extensive studies reported that alterations in n3 fatty acids, especially DHA and EPA are associated with alteration of photo-transduction efficiency, which in turn affect ERG responses [43, 44]. Therefore, we speculated that altered FA composition in the retinas of the RESV-treated mice likely helped maintain the ERG a- and b- wave amplitude. In our experiments, we found that light damage led to the increase of saturated FA, monoenoic FA, n6 PUFA and n6/n3 ratio (Table 1, SAMP1 vs SAMP8+LD). Even though the retinas of SAMP8 mice with RESV treatment still had lower DHA, RESV suppressed the levels of n6 PUFA as there is no difference in n6/n3 ratio between the two groups of SAMP1 and SAMP8+LD+RESV (Table 1). Studies on the protective effect of DHA for retinal function are controversial. Li and Anderson et al showed that DHA is not beneficial for the treatment of retinal degeneration in the animal model of Stargardts-like macular dystrophy [45]. Very long chain PUFAs are FAs of the n3 and n6 series with greater than C26 carbons. These unique groups of FAs are found mostly in the retina, brain, testis, and spermatozoa [46]. Ongoing studies have reported reduced levels of very long chain polyunsaturated FAs in aging retinas and in retinas of donor eyes of patients with a history of AMD [47]. We also noticed that light damage resulted in decrease in very long chain n3 PUFA (32:6n3, 34:5n3 and 34:6n3) and that RESV may maintain the levels of very long chain PUFA. However, there was no significant difference in their alteration due to the limited number of samples.

In conclusion, RESV administration is able to protect both the retinal structure and function of SAMP8 mice from severe light exposure via increasing expression of neuroprotective factors, as well as decreasing expression of pIκB. In the light of these results, RESV may be a beneficial agent that can exert a protective effect against light-induced retinal damage associated with aging. This study suggests that RESV may maintain the morphology and function of retina suffering from oxidative and/or inflammatory stress in aging retinal diseases including AMD and DRP.

## MATERIALS AND METHODS

### Animals and treatment

One-month-old male SAMP8 and SAMR1 mice were purchased from Nanjing qingzilan Co. Ltd. (Nanjing, China). All animals were used according to the guidelines of ethical committee of experimental animal care at Sichuan Provincial People’s Hospital of China. Mice were housed in an air-conditioned room with a 12h light/dark cycle, a constant temperature of 22 ± 2 °C, and relative humidity of 65 ± 15%. At 3 months old, the SAMP8 mice were randomly assigned to two experimental dietary groups: one untreated group and one RESV treatment group, each group contained 10 animals (20 eyes). Male SAMR1 mice (n=20 eyes), which showed normal characteristics, were used as the external control. Resveratrol was purchased from Actafarma (Madrid, Spain) and administered to the animals at a dose of 5 mg/kg/day. As mentioned in a previous study, water consumption of individual animals was estimated to be 0.8mL/10g body weight/day, which implied a water intake of about 2.5mL/day for each mouse of 3 months of age weighing around 30g [6]. Therefore, a solution of 6mg/100mL was prepared, which consisted of 0.15mg in the estimated 2.5mL of water/day. Resveratrol was dissolved in absolute ethanol and added to the drinking water to a final ethanol concentration of 0.066%. Water bottles were covered with aluminium foil to be protected from light, and the drinking fluid was changed every day. Non-treated animals received 0.1% alcohol in tap water.

After 30 days of feeding, both untreated and RESV treatment groups were exposed to intense bright light at 2,700 lux for 4h (acute light stress). After intense light exposure, these two groups were returned to their native dim cyclic light and fed their respective diets. Retinal function was measured with electroretinography (ERG) after one week of light damage. Then all the mice were euthanized with CO_2_ from a compressed gas tank in an euthanization chamber followed by cervical dislocation, and their eyeballs were harvested for retinal histological analysis.

### Electroretinography (ERG)

Flash ERGs were recorded with an ERG recording system (Diagnosys, Littleton, MA); for additional details refer to previous publications [48] [14]. Mice were maintained in total darkness overnight and were prepared for ERG recording in dim red light. Each mouse was anesthetized with ketamine (120mg/kg bodyweight intramuscularly) and xylazine (6mg/kg bodyweight, intramuscularly). One drop of 10% phenylephrine was applied to the cornea to dilate the pupil, and one drop of 0.5% proparacaine HCl was applied for local anesthesia. A reference electrode was positioned in the mouth and a ground electrode on the foot, and the mouse was placed inside a Ganzfeld illuminating sphere with a gold electrode placed on the cornea. Five increasing strobe flashes were used starting at an intensity of −5 followed by −3, -1, 1and 1.2 log cd.s/m^2^, and the ERG responses from both eyes were recorded to assess rod photoreceptor function (scotopic ERG). To evaluate cone function (photopic ERG), the mice were light adapted to 2 minutes exposure to a 32 cd/m^2^ background in the Ganzfeld to saturate the rod response. ERG recording was conducted with a brief white flash intensity ranging from -0.53 to +2.0 log cd.s/m^2^ flash. The flicker response was taken with 3Hz, 10Hz and 15Hz light flicks. The responses were computer averaged and recorded at 3–60 s intervals depending on flash intensity. For recording Short-wave-sensitive cone (S-cone)-mediated and middle-wave-sensitive cone (M-cone)-mediated ERG response, the mice were first light-adapted for 2 min in green light with 20cd/m^2^ intensity. ERG was recorded by alternating green and UV flashes. The intensities ranged from 0.1671×10^4^ to 55.7×10^4^ photon/μm^2^ for green flashes and from 0.0113×10^4^ to 33.9×10^4^ photon/μm^2^ for UV flashes, with a background green illumination of 20 cd/m^2^. The amplitude of the a-wave was measured from the pre-stimulus baseline to the a-wave trough. The amplitude of the b-wave was measured from the trough of the a-wave to the peak of the b-wave.

### Histological analysis

Mice were euthanized with CO_2_ and the eyes were enucleated and marked with a green dye on dorsal surface to mark the superior hemisphere. Eyes were then fixed in PERFIX (20% isopropanol, 2% trichloroacetic acid, 4% paraformaldehyde, and 2% zinc chloride) overnight and placed in 70% ethanol at room temperature for 4 days followed by embedding in paraffin. Sagittal sections through the center of the eye, including the optic disc, were cut at 5 μm thickness. After hematoxylin and eosin (H&E) staining, rows of photoreceptor nuclei were counted using light microscopy at nine equidistant points beginning at the optic nerve head toward both the inferior and superior retinal hemispheres.

### Real-time PCR

mRNA levels of selected genes were measured using real-time PCR with cDNA extracted from retinas as templates. Primers (Table 2) were designed using Primer Quest software (Integrated DNA Technologies, Coralville, IA, USA) spanning the intron–exon boundary to amplify the corresponding mRNAs without amplifying potentially contaminating genomic DNA. Real-time PCR was carried out with the SYBR green PCR master mix (Bio-Rad Lab., Hercules, CA, USA) using the MyiQ Single-Color Real-Time PCR detection system (Bio-Rad Lab., Hercules, CA, USA) according to the manufacturer’s instructions. Electrophoresis of PCR products was performed to identify that a single band of the correct size had been amplified.

**Table 2:** Sequence of the primers used for RT-PCR

Gene	Sequence
LIF	F:5’-TGAGATGCAGGGATTGTGCCCTTA-3’
	R:5’-AAATGAAGAGAGCATTGGCGCTGC-3’
CNTF	F:5’-AGAGCAATCACCTCTGACCCTTCA-3’
	R:5’-ATCTCACTCCAGCGATCAGTGCTT-3’
BDNF	F:5’-AGAAGGTTCGGCCCAACGAAGAAA-3’
	R:5’-GACATGTTTGCGGCATCCAGGTAA-3’
OSM	F:5’-TTTGACCCTCAGTCTCCTCATCCT-3’
	R:5’-AGGGCTCCAAGAGTGATTCTGTGT-3’
CT-1	F:5’-AAGACCACCAGACTGACTCCTCAA-3’
	R:5’-GCTGCACGTATTCCTCCAGAAGTT-3’
CLC	F:5’- ACGAGCCTGACTTCAATCCTCCT-3’
	R:5’- ACGCAAGTAACACAGGAGGTGACT-3’
RPL19	F:5’-TCACAGCCT-GTACCTGAAGG-3’
	R:5’-TCGTGCTTCCTTGGTCTTAG-3’

### Western blots

Retinas were harvested immediately after animals were euthanized and homogenized in a lysis buffer (50mM Tris–HCl (pH=7.5), 150mM NaCl, 5mM EDTA, 1% (v/v) NP-40, 5% (v/v) glycerol, and protease inhibitor cocktail (Calbiochem, San Diego, CA). Protein content was measured using BCA protein assay (Pierce, Rutherford, IL). Total protein from each sample (15μg) was electrophoresed on 4–20% gradient SDS polyacrylamide gels (Invitrogen, Carlsbad, CA), and transferred to nitrocellulose membranes (Bio-Rad, Hercules, CA). The membranes were incubated in blocking buffer [5% BSA in TBST (20mMTris–HCl, pH 7.5, 100mMNaCl, and 0.1% Tween-20)] for 1 h at room temperature, and then incubated overnight at 4°C with rabbit polyclonal anti-phospho-IκBα antibody (#2859, Cell Signaling Technology, Beverly, MA), in blocking buffer, followed by 1-hour incubation at room temperature with HRP-conjugated goat anti-rabbit secondary antibody (Amersham Biosciences, Piscataway, NJ). Signals were visualized using Super Signal West Dura extended duration substrate (Pierce, Rutherford, IL) and quantified by conventional digital image analysis using an Image Station 4000R (Eastman Kodak, Rochester, NY). Blots were stripped and reprobed with rabbit polyclonal anti-β actin (ab6276, Abcam) followed by appropriate secondary antibodies for quantification of bands.

### Retinal fatty acid analysis

Retinas were harvested from all the experimental mice and frozen immediately in liquid nitrogen. Retinal lipids were subsequently extracted following the method of Bligh Dyer technique [49] with minor modification [50]. The purified lipid extract was stored under nitrogen until used. To the purified lipid extracts, 50nmol of 15:0, 17:0, 23:0 each were added as internal standards. One milliliter of 16.6% concentrated HCl in methanol was then added and the tubes were sealed under N2 with Teflon-lined caps and heated at 100°C overnight. The tubes were cooled on ice and FA methyl esters (FAMEs) were extracted and processed as previously reported [51].

FAMEs were identified using an Agilent Technologies 7890A gas chromatograph (GC) with a 5975C inert XL mass spectrometer (MS) detector (Agilent Technologies; Santa Clara, CA). The GC-MS was operated in the electron impact total-ion and single-ion monitoring (SIM) modes. The injection volume was 1μl and the inlet, held at 280°C, was set to pulsed splitless mode. An Agilent Technologies DB-23 column (60m × 0.32mm × 0.25μm) was used with a helium carrier gas flow rate of 1.9 ml/min. The oven temperature began at 130°C for 1.0 min, was ramped to 170°C at 6.8°C/min, and then ramped to 215°C at 2.9°C/min. After holding at 215°C for 11.4 min, the oven was ramped to 230°C at 42°C/min and held for 9.6 min. The oven was then ramped to 290°C at 10°C/min and held for 14.4 min. The MS transfer line, ion source, and quadrupole temperatures were 290°C, 230°C, and 150°C, respectively. The PUFAs were identified by using the m/z ratios 79.1, 108.1, and 150.1 in SIM mode and the full scan mass spectra in total-ion mode. FAMEs were quantified using an Agilent Technologies 6890N GC with flame ionization detector (FID). Sample concentrations were determined by comparison to internal standards 15:0, 17:0, 23:0, and 30:3n6. The injection volume was 1 μl and the inlet, held at 280°C, was set to pulsed split mode (10:1 ratio). An Agilent Technologies DB-23 column (60m×0.32mm×0.25μm) was used with a hydrogen carrier gas constant pressure of 13.1 psi. The oven temperature began at 130°C for 0.8 min, was ramped to 170°C at 8.2°C/min then ramped to 215°C at 3.5°C/min. After holding at 215°C for 9.5 min, the oven was ramped to 230°C at 50°C/min and held for 8 min. The oven was then ramped to 290°C at 12.0°C/min and held for 12 min. The FID was held at 290°C.

### Statistical analysis

The statistical analysis was performed using GraphPad Prism 6 (GraphPad Software, La Jolla, CA). Results of ERG and HE are expressed as Mean±Standard Error (SE). Since there are only 3 or 4 samples in RT-PCR, western blots and fatty acid analysis, those results are expressed as Mean±Standard Deviation (SD). Differences between two groups were assessed using either paired or unpaired t-tests, while differences between more than two groups were assessed using Multivariate ANOVA followed by posthoc Scheffe test. A p value less than 0.05 was considered significant.
